# (1S*,5R*)-9-Phenyl-9-aza­bicyclo­[3.3.1]nonan-3-one

**DOI:** 10.1107/S1600536812020065

**Published:** 2012-05-12

**Authors:** Zhen-ju Jiang, Qi He, Zhen Li, Zhou-yu Wang

**Affiliations:** a Department of Pharmaceutics Engineering, Xihua University, Chengdu 610039, People’s Republic of China; b Research Institute of Natural Gas Economy, Petrochina Oil and Gas Field Company, Chengdu 610051, People’s Republic of China

## Abstract

In the title compound, C_14_H_17_NO, the piperidinone and piperidine rings both adopt a chair conformation. The chiral crystals were obtained from a racemic reaction product *via* spontaneous resolution.

## Related literature

For the synthesis, see: Zhang (2003 ▶). For applications of the compound, see: Vernekar *et al.* (2010 ▶); Lazny *et al.* (2011 ▶). For puckering analysis, see: Cremer & Pople (1975 ▶).
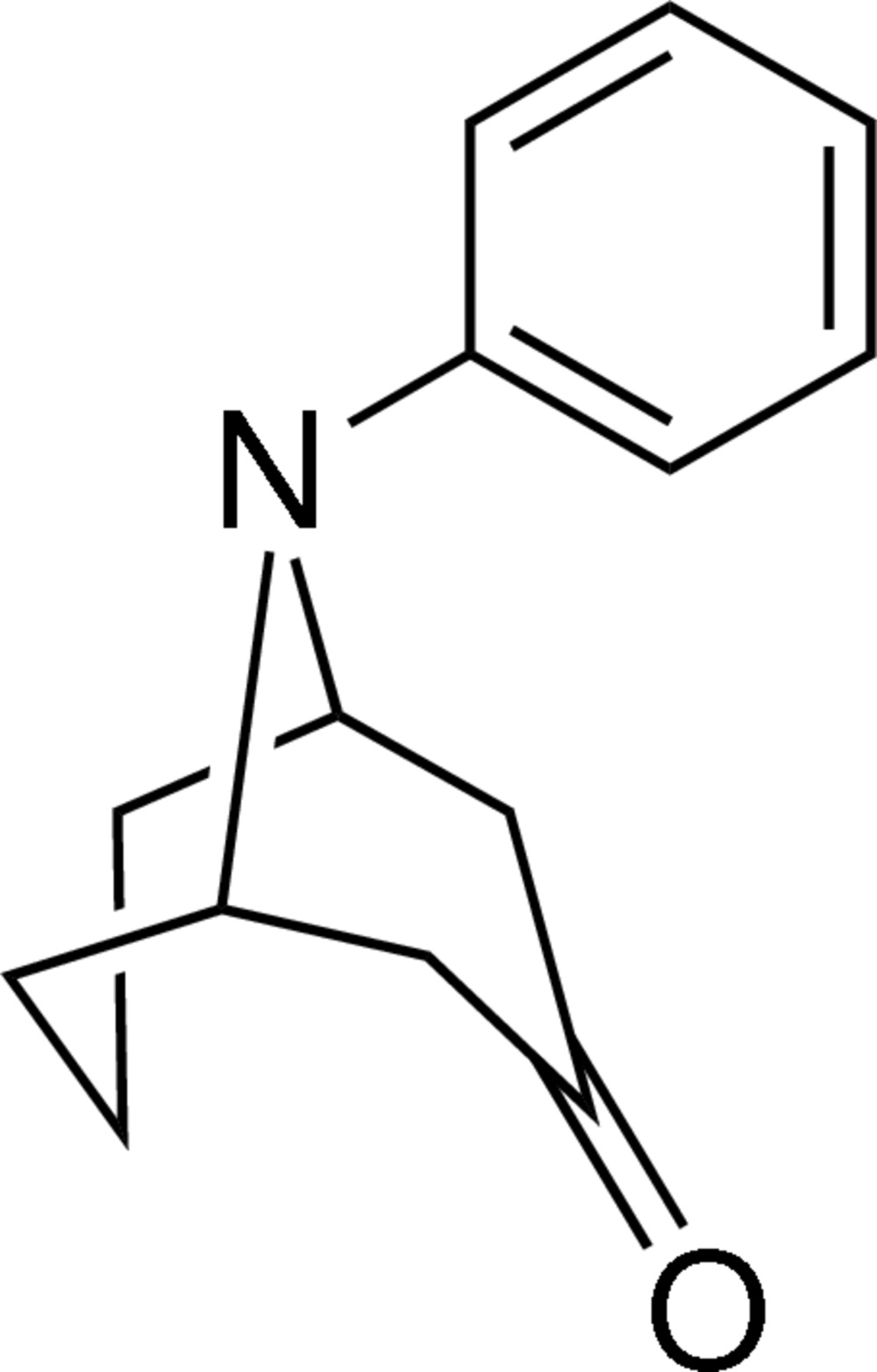


## Experimental

### 

#### Crystal data

C_14_H_17_NO
*M* *_r_* = 215.29
Orthorhombic, 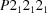

*a* = 9.4028 (3) Å
*b* = 10.2524 (5) Å
*c* = 12.0473 (6) Å
*V* = 1161.38 (9) Å^3^
*Z* = 4
Mo *K*α radiation
μ = 0.08 mm^−1^
*T* = 293 K
0.40 × 0.40 × 0.35 mm

#### Data collection

Agilent Xcalibur Eos diffractometer
Absorption correction: multi-scan (*ABSPACK* in *CrysAlis PRO*; Agilent, 2011 ▶) *T*_min_ = 0.918, *T*_max_ = 1.000
3285 measured reflections
2218 independent reflections
1722 reflections with *I* > 2σ(*I*)
*R*_int_ = 0.015

#### Refinement

*R*[*F* ^2^ > 2σ(*F* ^2^)] = 0.043
*wR*(*F* ^2^) = 0.092
*S* = 1.02
2218 reflections
145 parameters
H-atom parameters constrained
Δρ_max_ = 0.12 e Å^−3^
Δρ_min_ = −0.13 e Å^−3^

### 

Data collection: *CrysAlis PRO* (Agilent, 2011 ▶); cell refinement: *CrysAlis PRO*; data reduction: *CrysAlis PRO*; program(s) used to solve structure: *SHELXS97* (Sheldrick, 2008 ▶); program(s) used to refine structure: *SHELXL97* (Sheldrick, 2008 ▶); molecular graphics: *OLEX2* (Dolomanov *et al.*, 2009 ▶); software used to prepare material for publication: *OLEX2*.

## Supplementary Material

Crystal structure: contains datablock(s) global, I. DOI: 10.1107/S1600536812020065/fy2049sup1.cif

Structure factors: contains datablock(s) I. DOI: 10.1107/S1600536812020065/fy2049Isup3.hkl

Supplementary material file. DOI: 10.1107/S1600536812020065/fy2049Isup4.cdx

Supplementary material file. DOI: 10.1107/S1600536812020065/fy2049Isup4.cml

Additional supplementary materials:  crystallographic information; 3D view; checkCIF report

## Figures and Tables

**Fig. 1.**
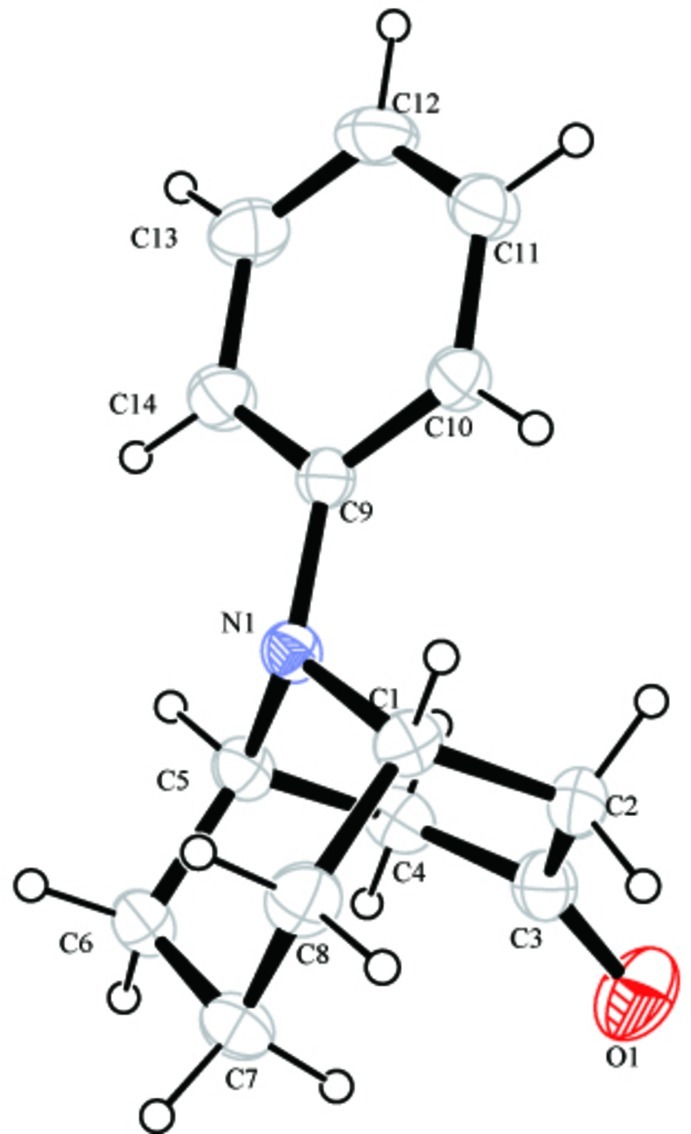
The molecular structure of the title compound.

**Fig. 2.**
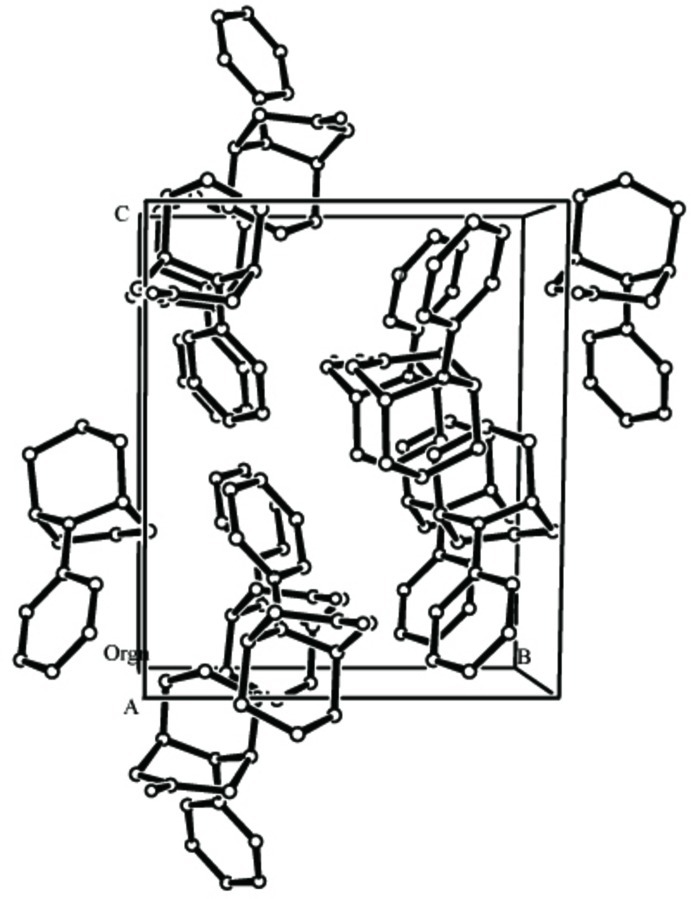
A packing diagram for the title compound.

## supplementary crystallographic information

### Comment

The compound 1*S**,5*R**-9-phenyl-9-aza-bicyclo[3.3.1]nonan-3-one is an important intermediate for synthesizing granisetron derivatives. The bicyclic skeleton of 9-azabicyclo[3.3.1]nonane is a key substructure of a variety of bioactive compounds (Vernekar *et al.*, 2010; Lazny *et al.*, 2011). The racemic title compound was synthesized by the Mannich reaction and spontaneous resolution occurred on recrystallization from a mixture of ethyl acetate and petroleum ether.

In the title structure the N1/C1—C5 piperidinone ring adopts a chair conformation with puckering parameters (Cremer & Pople, 1975): Q = 0.5159 (3) Å, θ = 158.26 (3)° and φ = 173.0692 (12)°. The N1/C1/C8—C5 piperidine ring has a chair conformation, too [Q = 0.5727 (3) Å, θ = 7.74 (12)° and φ = 23.8669 (13)°]. The relative configuration of C1 and C5 is S*, *R** respectively.

### Experimental

To a stirred solution of glutaraldehyde (1.32 ml, 5 mmol) and aniline (0.55 ml,6 mmol) in water (10 ml), 3-oxopentanedioic acid (0.88 g,6 mmol) was added. The mixture was stirred overnight at room temperature. Then the pH was adjusted to 5 with aq. HCl and the mixture was refluxed for another one hour. Then sodium hydroxide was added to increase the pH to 9. The mixture was extracted with ethyl-acetate. The combined extract was dried over anhydrous MgSO_4_ and evaporated *in vacuo*. The residue was purified through column chromatography on silica gel (eluent: hexane/EtOAc = 4/1) to give 9-phenyl-9-aza-bicyclo[3.3.1]nonan-3-one. Then the racemic mixture was crystallized from a solution in a 1:10 (*v*/*v*) mixture of ethyl acetate and petroleum ether to produce the title compound.

### Refinement

All H atoms were positioned geometrically and treated using a riding model, fixing the bond lengths at 0.97 Å for aliphatic CH, 0.98 Å for CH_2_ and 0.93 Å for aromatic CH groups, respectively. The displacement parameters of the H atoms were constrained with *U*_iso_(H) = 1.2*U*_eq_(C). In the absence of significant anomalous scattering effects, the absolute configuration is not determined.

### Crystal data

**Table:**

C_14_H_17_NO	*D*_x_ = 1.231 Mg m^−^^3^
*M**_r_* = 215.29	Mo *K*α radiation, λ = 0.7107 Å
Orthorhombic, *P*2_1_2_1_2_1_	Cell parameters from 1237 reflections
*a* = 9.4028 (3) Å	θ = 2.9–29.0°
*b* = 10.2524 (5) Å	µ = 0.08 mm^−^^1^
*c* = 12.0473 (6) Å	*T* = 293 K
*V* = 1161.38 (9) Å^3^	Block, colourless
*Z* = 4	0.40 × 0.40 × 0.35 mm
*F*(000) = 464	

### Data collection

**Table:**

Agilent Xcalibur Eos diffractometer	2218 independent reflections
Radiation source: Enhance (Mo) X-ray Source	1722 reflections with *I* > 2σ(*I*)
Graphite monochromator	*R*_int_ = 0.015
Detector resolution: 16.0874 pixels mm^-1^	θ_max_ = 26.4°, θ_min_ = 2.9°
ω scans	*h* = −11→7
Absorption correction: multi-scan (ABSPACK in *CrysAlis PRO*; Agilent, 2011)	*k* = −9→12
*T*_min_ = 0.918, *T*_max_ = 1.000	*l* = −8→15
3285 measured reflections	

### Refinement

**Table:**

Refinement on *F*^2^	Primary atom site location: structure-invariant direct methods
Least-squares matrix: full	Secondary atom site location: difference Fourier map
*R*[*F*^2^ > 2σ(*F*^2^)] = 0.043	Hydrogen site location: inferred from neighbouring sites
*wR*(*F*^2^) = 0.092	H-atom parameters constrained
*S* = 1.02	*w* = 1/[σ^2^(*F*_o_^2^) + (0.0394*P*)^2^] where *P* = (*F*_o_^2^ + 2*F*_c_^2^)/3
2218 reflections	(Δ/σ)_max_ < 0.001
145 parameters	Δρ_max_ = 0.12 e Å^−^^3^
0 restraints	Δρ_min_ = −0.13 e Å^−^^3^

### Special details

**Table:**

Geometry. All e.s.d.'s (except the e.s.d. in the dihedral angle between two l.s. planes) are estimated using the full covariance matrix. The cell e.s.d.'s are taken into account individually in the estimation of e.s.d.'s in distances, angles and torsion angles; correlations between e.s.d.'s in cell parameters are only used when they are defined by crystal symmetry. An approximate (isotropic) treatment of cell e.s.d.'s is used for estimating e.s.d.'s involving l.s. planes.
Refinement. Refinement of *F*^2^ against ALL reflections. The weighted *R*-factor *wR* and goodness of fit *S* are based on *F*^2^, conventional *R*-factors *R* are based on *F*, with *F* set to zero for negative *F*^2^. The threshold expression of *F*^2^ > σ(*F*^2^) is used only for calculating *R*-factors(gt) *etc*. and is not relevant to the choice of reflections for refinement. *R*-factors based on *F*^2^ are statistically about twice as large as those based on *F*, and *R*- factors based on ALL data will be even larger.

### Fractional atomic coordinates and isotropic or equivalent isotropic displacement parameters (Å^2^)

**Table:**

	*x*	*y*	*z*	*U*_iso_*/*U*_eq_	
O1	−0.58709 (17)	−0.01586 (16)	−0.68342 (15)	0.0838 (6)	
N1	−0.19284 (14)	−0.18245 (14)	−0.65084 (12)	0.0368 (4)	
C1	−0.31907 (18)	−0.26416 (18)	−0.63463 (16)	0.0409 (5)	
H1	−0.2985	−0.3506	−0.6653	0.049*	
C2	−0.4480 (2)	−0.20832 (19)	−0.69713 (16)	0.0472 (5)	
H2A	−0.4367	−0.2265	−0.7757	0.057*	
H2B	−0.5329	−0.2532	−0.6721	0.057*	
C3	−0.4692 (2)	−0.0642 (2)	−0.68231 (16)	0.0510 (6)	
C4	−0.3376 (2)	0.01633 (18)	−0.67070 (17)	0.0489 (5)	
H4A	−0.3618	0.0971	−0.6332	0.059*	
H4B	−0.3032	0.0386	−0.7442	0.059*	
C5	−0.2173 (2)	−0.05077 (18)	−0.60634 (17)	0.0412 (5)	
H5	−0.1302	0.0001	−0.6174	0.049*	
C6	−0.2468 (2)	−0.0573 (2)	−0.48223 (17)	0.0494 (5)	
H6A	−0.1612	−0.0854	−0.4442	0.059*	
H6B	−0.2703	0.0293	−0.4556	0.059*	
C7	−0.3672 (2)	−0.1497 (2)	−0.45334 (16)	0.0522 (6)	
H7A	−0.3705	−0.1626	−0.3736	0.063*	
H7B	−0.4571	−0.1122	−0.4766	0.063*	
C8	−0.3452 (2)	−0.2797 (2)	−0.51068 (17)	0.0510 (5)	
H8A	−0.4286	−0.3338	−0.4994	0.061*	
H8B	−0.2646	−0.3239	−0.4774	0.061*	
C9	−0.11420 (18)	−0.19480 (18)	−0.75009 (15)	0.0363 (4)	
C10	−0.1472 (2)	−0.28634 (19)	−0.83142 (16)	0.0435 (5)	
H10	−0.2261	−0.3400	−0.8221	0.052*	
C11	−0.0647 (2)	−0.2989 (2)	−0.92593 (16)	0.0537 (6)	
H11	−0.0896	−0.3602	−0.9794	0.064*	
C12	0.0534 (2)	−0.2223 (2)	−0.94206 (16)	0.0597 (6)	
H12	0.1084	−0.2308	−1.0058	0.072*	
C13	0.0880 (2)	−0.1329 (2)	−0.8617 (2)	0.0605 (6)	
H13	0.1680	−0.0807	−0.8712	0.073*	
C14	0.0069 (2)	−0.1187 (2)	−0.76732 (18)	0.0497 (5)	
H14	0.0333	−0.0574	−0.7142	0.060*	

### Atomic displacement parameters (Å^2^)

**Table:**

	*U*^11^	*U*^22^	*U*^33^	*U*^12^	*U*^13^	*U*^23^
O1	0.0585 (9)	0.0818 (13)	0.1110 (16)	0.0252 (10)	−0.0147 (10)	0.0074 (12)
N1	0.0385 (8)	0.0357 (8)	0.0362 (9)	−0.0040 (7)	0.0001 (7)	−0.0009 (7)
C1	0.0411 (10)	0.0361 (10)	0.0455 (12)	−0.0027 (9)	0.0024 (9)	0.0022 (9)
C2	0.0401 (10)	0.0537 (12)	0.0478 (13)	−0.0053 (10)	−0.0056 (9)	−0.0027 (11)
C3	0.0514 (12)	0.0589 (13)	0.0428 (13)	0.0091 (12)	−0.0071 (10)	0.0064 (11)
C4	0.0646 (13)	0.0389 (10)	0.0431 (12)	0.0059 (11)	0.0016 (11)	0.0032 (9)
C5	0.0461 (11)	0.0371 (10)	0.0405 (11)	−0.0071 (9)	0.0010 (9)	−0.0024 (9)
C6	0.0536 (11)	0.0577 (13)	0.0370 (12)	0.0021 (11)	−0.0035 (10)	−0.0052 (11)
C7	0.0590 (13)	0.0634 (13)	0.0343 (11)	−0.0013 (11)	0.0053 (10)	0.0057 (10)
C8	0.0501 (12)	0.0536 (12)	0.0494 (12)	−0.0058 (11)	0.0045 (10)	0.0128 (11)
C9	0.0371 (9)	0.0379 (10)	0.0340 (10)	0.0052 (9)	−0.0020 (8)	0.0040 (9)
C10	0.0425 (10)	0.0446 (11)	0.0434 (11)	0.0035 (10)	−0.0026 (9)	−0.0023 (10)
C11	0.0581 (13)	0.0581 (14)	0.0450 (13)	0.0129 (12)	−0.0016 (11)	−0.0081 (11)
C12	0.0618 (14)	0.0737 (16)	0.0436 (13)	0.0145 (13)	0.0173 (12)	0.0027 (12)
C13	0.0533 (13)	0.0651 (15)	0.0630 (16)	−0.0072 (12)	0.0156 (12)	0.0051 (13)
C14	0.0495 (11)	0.0528 (13)	0.0467 (13)	−0.0078 (11)	0.0043 (10)	−0.0023 (10)

### Geometric parameters (Å, º)

**Table:**

O1—C3	1.215 (2)	C6—C7	1.517 (3)
N1—C1	1.466 (2)	C7—H7A	0.9700
N1—C5	1.471 (2)	C7—H7B	0.9700
N1—C9	1.412 (2)	C7—C8	1.516 (3)
C1—H1	0.9800	C8—H8A	0.9700
C1—C2	1.537 (3)	C8—H8B	0.9700
C1—C8	1.522 (3)	C9—C10	1.392 (3)
C2—H2A	0.9700	C9—C14	1.396 (3)
C2—H2B	0.9700	C10—H10	0.9300
C2—C3	1.501 (3)	C10—C11	1.384 (3)
C3—C4	1.494 (3)	C11—H11	0.9300
C4—H4A	0.9700	C11—C12	1.374 (3)
C4—H4B	0.9700	C12—H12	0.9300
C4—C5	1.534 (3)	C12—C13	1.373 (3)
C5—H5	0.9800	C13—H13	0.9300
C5—C6	1.522 (3)	C13—C14	1.376 (3)
C6—H6A	0.9700	C14—H14	0.9300
C6—H6B	0.9700		
			
C1—N1—C5	110.46 (14)	C7—C6—C5	112.88 (17)
C9—N1—C1	119.07 (15)	C7—C6—H6A	109.0
C9—N1—C5	118.22 (14)	C7—C6—H6B	109.0
N1—C1—H1	107.9	C6—C7—H7A	109.6
N1—C1—C2	111.11 (15)	C6—C7—H7B	109.6
N1—C1—C8	108.76 (15)	H7A—C7—H7B	108.2
C2—C1—H1	107.9	C8—C7—C6	110.08 (16)
C8—C1—H1	107.9	C8—C7—H7A	109.6
C8—C1—C2	113.10 (16)	C8—C7—H7B	109.6
C1—C2—H2A	108.7	C1—C8—H8A	109.2
C1—C2—H2B	108.7	C1—C8—H8B	109.2
H2A—C2—H2B	107.6	C7—C8—C1	112.14 (16)
C3—C2—C1	114.39 (17)	C7—C8—H8A	109.2
C3—C2—H2A	108.7	C7—C8—H8B	109.2
C3—C2—H2B	108.7	H8A—C8—H8B	107.9
O1—C3—C2	121.5 (2)	C10—C9—N1	122.68 (16)
O1—C3—C4	122.1 (2)	C10—C9—C14	117.02 (18)
C4—C3—C2	116.42 (18)	C14—C9—N1	120.20 (17)
C3—C4—H4A	108.7	C9—C10—H10	119.4
C3—C4—H4B	108.7	C11—C10—C9	121.13 (19)
C3—C4—C5	114.20 (16)	C11—C10—H10	119.4
H4A—C4—H4B	107.6	C10—C11—H11	119.5
C5—C4—H4A	108.7	C12—C11—C10	121.1 (2)
C5—C4—H4B	108.7	C12—C11—H11	119.5
N1—C5—C4	110.04 (15)	C11—C12—H12	120.9
N1—C5—H5	108.0	C13—C12—C11	118.28 (19)
N1—C5—C6	110.25 (16)	C13—C12—H12	120.9
C4—C5—H5	108.0	C12—C13—H13	119.3
C6—C5—C4	112.44 (16)	C12—C13—C14	121.5 (2)
C6—C5—H5	108.0	C14—C13—H13	119.3
C5—C6—H6A	109.0	C9—C14—H14	119.5
C5—C6—H6B	109.0	C13—C14—C9	121.0 (2)
H6A—C6—H6B	107.8	C13—C14—H14	119.5
			
O1—C3—C4—C5	146.4 (2)	C5—N1—C1—C2	61.85 (19)
N1—C1—C2—C3	−46.2 (2)	C5—N1—C1—C8	−63.28 (19)
N1—C1—C8—C7	58.9 (2)	C5—N1—C9—C10	−142.12 (17)
N1—C5—C6—C7	−54.0 (2)	C5—N1—C9—C14	41.6 (2)
N1—C9—C10—C11	−177.64 (17)	C5—C6—C7—C8	49.0 (2)
N1—C9—C14—C13	177.52 (18)	C6—C7—C8—C1	−51.6 (2)
C1—N1—C5—C4	−63.47 (19)	C8—C1—C2—C3	76.4 (2)
C1—N1—C5—C6	61.12 (19)	C9—N1—C1—C2	−79.9 (2)
C1—N1—C9—C10	−3.3 (2)	C9—N1—C1—C8	155.02 (16)
C1—N1—C9—C14	−179.62 (16)	C9—N1—C5—C4	78.60 (18)
C1—C2—C3—O1	−148.4 (2)	C9—N1—C5—C6	−156.81 (15)
C1—C2—C3—C4	34.0 (2)	C9—C10—C11—C12	0.7 (3)
C2—C1—C8—C7	−65.0 (2)	C10—C9—C14—C13	1.0 (3)
C2—C3—C4—C5	−35.9 (2)	C10—C11—C12—C13	0.2 (3)
C3—C4—C5—N1	49.8 (2)	C11—C12—C13—C14	−0.4 (3)
C3—C4—C5—C6	−73.5 (2)	C12—C13—C14—C9	−0.2 (3)
C4—C5—C6—C7	69.2 (2)	C14—C9—C10—C11	−1.2 (3)

## References

[bb1] Agilent (2011). *CrysAlis PRO* Agilent Technologies UK Ltd, Yarnton, Oxfordshire, England.

[bb2] Cremer, D. & Pople, J. A. (1975). *J. Am. Chem. Soc.* **97**, 1354–1358.

[bb3] Dolomanov, O. V., Bourhis, L. J., Gildea, R. J., Howard, J. A. K. & Puschmann, H. (2009). *J. Appl. Cryst.* **42**, 339–341.

[bb4] Lazny, R., Wolosewicz, K., Zielinska, P., Lipkowska, Z. U. & Kalicki, P. (2011). *Tetrahedron*, **67**, 9433–9439.

[bb5] Sheldrick, G. M. (2008). *Acta Cryst.* A**64**, 112–122. 10.1107/S0108767307043930 18156677

[bb6] Vernekar, S. K. V., Hallaq, H. Y., Clarkson, G., Thompson, A. J., Silvestri, L., Lummis, S. C. R. & Lochner, M. (2010). *J. Med. Chem.* **53**, 2324–2328. 10.1021/jm901827x PMC4166935 20146481

[bb7] Zhang, Y. (2003). CN Patent 1451660A.

